# Translational Perspectives on Binge-Eating: Insights from Animal Models

**DOI:** 10.1007/s11920-026-01661-9

**Published:** 2026-03-05

**Authors:** Estefania P. Azevedo, Sarah A. Stern

**Affiliations:** 1 Medical University of South Carolina, Charleston, SC USA; 2 Max Plank Florida Institute for Neuroscience, Max Planck Way, Jupiter, FL 33459 USA

**Keywords:** Binge-eating, Eating disorders, Animal models, Stress, neural circuits

## Abstract

**Purpose of Review:**

Binge-eating is a behavior that is both recognized in both binge-eating disorder and binge-subtypes of restrictive eating disorders. Binge-eating is distinct from obesity, although the two frequently co-occur. The purpose of this review is to examine animal models of binge like behavior independent of obesity as a phenotype and to examine recent findings.

**Recent Findings:**

Current research indicates three major precipitants of binge-like behavior in rodents, namely limited access to palatable food, limited access to food following stress, and cue-induced overconsumption. These models have enabled mechanistic studies, which highlight the role of several key brain regions and circuits in binge-eating.

**Summary:**

Taken together, these studies highlight the important role that preclinical models play in elucidating the mechanisms underlying binge-eating and to develop therapeutic interventions.

## Introduction

Binge-eating is a behavior defined in the DSM-5 [1] as the consumption of a large amount of food in a short period of time (usually around 2 h), in addition to lack of control during the binge episode. Binge-eating disorder (BED) is also associated with negative affect associated with the binge, eating rapidly and/or alone, eating until uncomfortably full, recurrent episodes over a 3–6 month period, and lack of compensatory behaviors such as purging, fasting or excessive exercise. Although binge-eating is the major diagnostic criteria for binge-eating disorder and is often associated with obesity [2], binge-eating behavior is also present in many forms of eating disorders which include compensatory behaviors, including bulimia nervosa and the binge/purge subtype of anorexia nervosa [1]. Binge eating is frequently comorbid with other psychiatric conditions, most notably mood disorders (e.g., major depressive disorder), anxiety disorders, and substance use disorders. This high degree of comorbidity complicates clinical studies, as overlapping symptoms such as negative affect, stress responsivity, and impulsivity may reflect shared or interacting mechanisms rather than processes specific to binge eating itself. In addition, comorbid diagnoses and their treatments can introduce substantial heterogeneity into patient samples, making it difficult to isolate binge-eating–specific neural, behavioral, or metabolic signatures [3–5]. Preclinical studies are therefore one factor that can help to disentangle the mechanisms that contribute to binge-eating in isolation from other comorbid factors [6–8]. Food intake is readily measurable in rodents, and the meal patterns are well established, enabling an assessment of changes that may relate to binge-eating. In this Review, we will highlight the three major categories of rodent models of binge-eating, with a particular emphasis on how they were first developed and recent advances. We will then discuss key neurobiological findings from these models. Lastly, we will suggest paths forward for maximizing potential and utilizing modern tools to gain new insights into binge-eating behavior.

### Models of Binge Eating Behaviors in Rodents

Models of binge-eating in rodents generally fall into 3 major categories: limited access to palatable food, limited access with stress, and cue induced eating. Each of these models has several variations, including the type of food made available, the precise timing of access, and the length of the protocol. Although we will not discuss each variation in detail, below we will capture the essential components of each category and assess how well they model the core features of BED: (1) Eating rapidly in the absence of hunger (2) Loss of control and (3) Negative emotional state. Although most animal models are unable to capture all three of these elements, important fundamental mechanisms can be learned even if only some of the components are there. We consider that the essential element to assess binge-eating disorders is the presence of the binge itself, and we therefore consider this to be a prerequisite component. Although studies of addiction and motivation on their own may also provide important insights for BED, we consider those outside the scope of this review. It is also critical to distinguish binge-eating, which occurs in the absence of hunger or past the satiety state, from increased consumption driven by homeostatic factors such as fasting or negative energy balance. Accordingly, only models that assess non-homeostatic food intake in a sated state are discussed and it is critical to examine the protocols to determine the metabolic state of the animals at the time of food intake assessments.

Existing mouse models may not always use the term “binge”, but sometimes “overconsumption”, “non-homeostatic food intake” and “compulsive intake.” Overconsumption and non-homeostatic food intake typically refer to eating past homeostatic needs and is often used to avoid conflation with BED. Compulsive intake is a construct used in human studies to assess eating along the parameters used to diagnose substance abuse [9], and also used in rodent studies to describe binge-like responding for food that occurs despite negative consequences. In relation to animal models of binge-eating, those terms will therefore be used synonymously in this Review.

#### Limited Access Models

At their core, many of the binge-eating models use limited access to palatable food, either with or without prior food restriction. Although palatable food consumption is not a diagnostic criteria for BED, it is a typical feature [10].

The first demonstration of the limited access model was by Corwin in 1998 [11], in which rats were given 2 h of access to vegetable shortening (e.g. high fat) for 3 days a week. Compared to rats who were given access to the shortening every day, mice ate significantly more during the access periods. Importantly in this model, chow was present for the duration of the protocol, demonstrating that binge-eating occurred independent of a caloric deficit. However, as the model progressed, rats learned to compensate by undereating following the binge-session. Thus, it is not entirely clear if the observed binge-like behavior is motivated at least partially by the decrease in intake on the prior day(s), but this may be true in humans as well [12, 13]. In a subsequent study, the same group modified the intermittent access group so that they were given more shortening access sessions in weeks 1 and 2, but less in weeks 3 and 4, ensuring that there were at least 4 days between access sessions, and enabling the animals to return to normal food intake patterns [14]. Interestingly, these rats still consumed significantly more during the last 2 sessions, demonstrating that the binge behavior persists even once decreased prior intake is removed. Animals with intermittent access also increased their motivation for shortening in a progressive ratio task, compared to daily access mice.

Similar demonstrations of limited access models have been successful with once-weekly 24-hour access to high fat-high sugar (HFHS) diet [15], and daily for 1 h [16], as well as with sugar alone [17].

Although the precise details of when highly palatable food (HPF) is given during these protocols varies, the core principle across all is that intermittent access to these foods promotes increased intake of those foods. Recently, one study [18] directly compared continuous, daily intermittent and 3x weekly intermittent HPF access and found that daily intermittent access produces the most robust binge-like behavior. Despite this, weight increased more in animals with continuous access to HPF. Thus, although binge-eating behavior is often associated with weight gain and obesity in the clinical population, this is not necessarily replicated in animal models.

It is challenging to directly measure loss of control and negative affect in the limited access model, although some studies have shown an increase in anxiety-like behavior following binge-like episodes, or binge-like behavior despite negative consequences [19–21], indicating the possible presence of negative affect.

#### Limited Access with Stress Models

Research in patients with binge-eating disorder have shown that binges often are directly preceded by negative mood, including sadness, depression, frustration and anger. In addition, converging research suggests that BED patients experience greater stress and are more sensitive to stressful events than healthy controls [22]. Thus, understanding how stress impacts binge-like behavior in rodents, and identifying the corresponding neural mechanisms is of high interest.

The first of these [23] paired cycles of food restriction and HPF + ad libitum chow access (similar to weight cycling [24]) with a foot shock to elicit stress. This model led rats to significantly increase HPF consumption as well as chow consumption following brief exposure to HPF. Subsequent models [25, 26] paired a similar restriction-cycle with intermittent access to HPF and either forced swim stress or a frustrative experience in which animals could see and smell the HPF but did not have access to it. Food restriction is not necessarily required for stress-induced binge-eating, as it can also be elicited in female mice with ad-libitum chow alongside limited access to HPF and frustrative stress [27]. Yet another recent model [28] demonstrated that early life stress, consisting of maternal separation, exacerbated binge-like eating in a limited access model with 1 day per week high fat diet (HFD). Interestingly, studies show that binge-episodes in patients are often provoked by negative feelings associated with interpersonal relationships, including loneliness. Accordingly, studies using chronic social defeat stress have shown that social stress leads to increased body weight and binge-like behavior [29, 30]. Similarly, another model showed that socially isolated mice exhibited increased consumption during the earlier part of the binge episode compared to group housed mice [31].

Like the limited access models, the precise details of these protocols may differ, but it seems generally clear that stress is a powerful factor in increasing binge-like behavior. One element to note, however, is that in these cases stress is typically acute and occurs at an isolated period, with the exception of chronic social defeat. It is unclear what effect chronic stress paradigms would have on binge-like behavior and whether it would be similar. Interestingly, acute stress models without associated alterations in food delivery typically results in weight loss (e.g. stress induced anorexia) [32, 33], suggesting that links to obesity may emerge as stress becomes chronic or when stress is accompanied by limited access to food.

#### Cue-Induced Models

Another set of models diverges significantly from the previous two and explores the idea that cues, which have been shown to powerfully control relapse behavior in drug addiction [34, 35], may play a similar role in bingeing behavior. Specifically, they test whether re-exposure to contexts and cues that are associated with food intake or hunger may promote binge-like episodes.

The notion that cues might potentiate feeding was first demonstrated by Henry Weingarten as early as the 1980’s when he showed that a cue preceding food delivery under periods of food deprivation then led to decreased latency to eat in a subsequent session when rats were sated [36]. Weingarten did not quantify the amount of food consumed, but Petrovich and colleagues later extended this task and demonstrated that rats significantly increased consumption following cue-food associations [37].

Contextual cues were also found to be sufficient to induce overconsumption past satiety states. In one model, rats were trained to consume HPF in a particular context, driving contextual associations with the food. When later placed back into that context with chow, rats displayed binge-like behavior of chow [38]. A similar study using sucrose showed increased sucrose consumption in an associated context even following sucrose-specific satiation [39]. However, subsequent studies showed that binge-like behavior could be elicited even when trained on normal chow in the context [40, 41], and even through negative reinforcement of just associating the context with a hunger state. Thus, regardless of whether the food is highly palatable or not, all models suggest that environmental cues can regulate feeding and may be a significant contributing factor in eliciting a binge episode.

### Limitations of Animal Models

There are several considerations when discussing the usefulness of animal models of binge-eating. At the outset, preclinical models can never be assumed as a substitute for human disease and ethological considerations must define the experiments and their interpretation. For example, binge-eating in patients is defined as occurring over a short period of time, typically around 2 h. This time frame would not be reasonable in a rodent model, where food intake is distributed more equally over the circadian cycle and typically occurs in a more fragmented pattern than in humans, partially owing to their high basal metabolic rate [42, 43]. Moreover, as mentioned previously, binge-eating disorder is defined by a loss of control (a perceived inability to control eating) during the binge-episode, but also with a sense of marked distress during and after the binge [44, 45]. These elements are more challenging to assess in animal models, but may be inferred from other tasks designed to test affect, motivation and approach/avoidance behaviors [46, 47]. Despite these caveats, animal models can provide important insights into the key mechanisms that drive binge-like behavior.

#### Loss of Control and Negative Affect

Although as mentioned above, we cannot truly assess loss of control or feelings of guilt and disgust in animals, it is possible to measure indicators of negative affect, as well as bingeing despite adverse consequences. For example, one model showed that mice exposed to a limited access plus foot shock model were willing to expose themselves to escalating levels of foot shock in order to receive a palatable food [21].

#### Relevance to Obesity

Although binge eating is often associated with obesity, most rodent models of binge-like intake do not produce measurable weight gain, in contrast to paradigms involving continuous access to a high-fat diet. This feature can be advantageous, as it enables the investigation of neural and behavioral mechanisms underlying binge eating independently of those driving obesity. Consistent with this distinction, binge-eating disorder can occur across a wide range of body weights, and binge-eating behaviors are also observed in eating disorders characterized by compensatory behaviors, in which net weight gain may not occur [1].

### Brain Mechanisms Governing binge-eating Behavior

#### *Hypothalamus*

The hypothalamus is a central regulator of feeding behavior, integrating internal metabolic signals with sensory and reward-related inputs to coordinate energy intake [48]. Distinct hypothalamic nuclei, including the arcuate nucleus, lateral hypothalamic area, paraventricular nucleus, and ventromedial hypothalamus, contain specialized neuronal populations that either promote or suppress feeding through different mechanisms [48]. These neurons monitor hormonal and nutrient cues, such as leptin, insulin, and ghrelin, and project to several brain regions, enabling the hypothalamus to adjust motivation for food and control meal size according to the body’s energetic needs. The hypothalamus also shapes palatable food seeking and binge-like intake through defined neuron classes and projections whose causal roles have been established with loss- and gain-of-function tools [49–51].

Binge eating can sometimes share similar mechanisms to homeostatic eating. For example, optogenetic or chemogenetic activation of agouti-related peptide/neuropeptide Y (AgRP/NPY) neurons in the arcuate nucleus rapidly drives voracious feeding even in sated mice [52–54], establishing these cells as potent promoters of intake and appetitive behavior, whereas silencing them reduces feeding. By contrast, pro-opiomelanocortin (POMC) neurons exert anorexigenic control as ablation of POMC neurons in the arcuate nucleus leads to hyperphagia [55, 56], indicating a mechanistic counterweight to AgRP drive. In a binge eating study, injections of hypothalamic peptides such as NPY and orexin-A in the nucleus accumbens significantly increased palatable, hedonic eating, while injections of cocaine- and amphetamine-related transcript (CART) reduced intake [57]. Indeed, many of the brain regions connected with the hypothalamus drive hedonic, binge eating and express a variety of receptors for hypothalamic molecules [48]. For example, in rats with binge eating induced by limited access to a high-fat sweet diet, CART expression increased in arcuate nucleus (ARC), lateral hypothalamus (LHA), shell area of the NAc, and PVT during binges [58]. Whether CART or other neuropeptide-expressing hypothalamic subpopulations play a direct role in binge eating episodes via these specific projections remains to be studied.

Beyond the ARC, LHA cell types bidirectionally regulate consumption and reward. Selective optogenetic stimulation of an inhibitory Vgat-expressing LHA subset enhances both appetitive and consummatory behaviors, while genetic ablation reduces them [59–61]. In contrast, LHA glutamatergic Vglut2 + neurons suppress feeding and drive aversion when activated, and their genetic ablation increases caloric intake [62, 63]. As part of the LHA Vglut2 + population, melanin-concentrating (MCH) neurons signal to promote palatable intake and motivation. MCH1 receptor antagonism reduces consumption of highly palatable condensed milk, and NAc shell MCH signaling increases feeding, suggesting another hypothalamus-accumbens neuropeptidergic route that enhances reward-driven eating [64, 65]. Disruption of the lateral hypothalamic MCH-ventral hippocampus pathway increases impulsivity in rodents, a key behavioral trait that predisposes to binge eating by weakening inhibitory control over palatable food intake [66]. Conversely, oxytocinergic mechanisms originating in hypothalamic nuclei act to restrain reward-seeking and sugar intake as intracerebroventricular oxytocin diminishes food seeking, impulsivity, and effortful choice for sucrose [67]. Another LHA peptide that plays an important role in feeding and motivation is neurotensin (NT). NT-expressing neurons in the LHA are activated by leptin and other anorectic cues and are essential for regulating energy balance, as their ablation increases adiposity and reduces locomotion, while their influence on body weight involves both orexin-dependent and orexin-independent mechanisms [68]. LHA NT neurons project directly to the ventral tegmental area (VTA) and modulates dopaminergic function, affecting reward-seeking and motivation [68–70].

The interplay between hypothalamic neuropeptides and neurotransmitters in binge-eating behaviors remain understudied, but studies suggest that the hypothalamus contains specialized neuronal populations that are important for several aspects of binge eating behavior, including motivation and impulsivity.

#### Nucleus Accumbens

The nucleus accumbens (NAc) is a central brain region located in the mesolimbic reward system and plays a crucial role in the development of binge eating by mediating the reinforcing properties of palatable food. It is primarily composed of GABAergic medium spiny neurons (MSNs), which are divided into two main populations: D1 receptor-expressing and D2 receptor-expressing neurons. These MSNs receive dense excitatory input from the prefrontal cortex, hippocampus, and amygdala, and project to downstream basal ganglia structures, such as the ventral pallidum and substantia nigra [71].

In rodent models, binge-like consumption of sugar and fat increases dopamine release in the NAc shell, which enhances reward learning and reinforces compulsive intake patterns [72, 73]. Repeated binge episodes sensitize dopamine D1 receptor-expressing MSNs, facilitating compulsive food seeking [74]. Importantly, bingeing on high-fat food leads to increased evoked dopamine release and reduced dopamine reuptake in the NAc, reflecting impaired dopamine transporter function and prolonged dopaminergic signaling that may enhance the salience of food cues [75]. Additionally, local activation of mu-opioid receptors in the NAc shell robustly promotes overconsumption of high-fat and sugary foods, implicating endogenous opioid signaling in hedonic feeding [76]. One study shows that modulation of prefrontal cortex (PFC) excitatory inputs to the nucleus accumbens (NAc), suppresses high fat intake while anterior paraventricular thalamus (PVT) inputs to the NAc promotes high fat intake [77]. Other studies reinforce the hypothesis of opioid signaling in the accumbens being important for binge eating behavior. Stimulation of nucleus accumbens µ-opioid receptors promotes binge-like eating, while concurrent glucagon-like peptide-1 (GLP-1) receptor activation suppresses this effect and GLP-1 receptor blockade prolongs it, demonstrating a direct interaction between opioid and GLP-1 signaling in regulating palatable food intake [78]. Female rats with limited, irregular access to chocolate develop binge-eating behaviors accompanied by increased striatal µ-opioid receptors, implicating dysregulated opioidergic signaling in binge-eating behavior in this model [79]. Another recent finding suggests another peptidergic mechanism for binge eating in the nucleus accumbens. Recent findings show that the neuropeptide pituitary adenylate cyclase-activating polypeptide (PACAP) increases the excitability of both D1- and D2-expressing MSNs in the NAc, suggesting a mechanism by which stress or neuropeptidergic modulation may lower the threshold for reward-driven food seeking [80]. This excitatory effect on both MSN subtypes may contribute to the maladaptive behaviors observed during binge episodes.

#### Insular Cortex

The insular cortex (IC) is a key hub integrating interoceptive, gustatory, and emotional signals that influence feeding behavior. It is primarily composed of glutamatergic pyramidal neurons in layers V/VI and receives inputs from the paraventricular thalamus (PVT), hypothalamus, basolateral amygdala, brainstem, and thalamus, while projecting to limbic and reward-related regions such as the nucleus accumbens (NAc), central amygdala (CeA), and ventral striatum [81, 82].

In rodent models, the IC plays a crucial role in overconsumption. Inhibition of the IC attenuates food cue reactivity and reduces consumption of palatable food, indicating that hyperactivity in this region contributes to maladaptive feeding [83, 84]. The IC also participates in associative learning of contextual food cues that drive non-homeostatic feeding in sated states [40]. Notably, a subset of Nos1-expressing neurons in the IC has been identified as critical mediators of top-down control of conditioned overconsumption, suggesting that distinct molecularly defined populations within the IC contribute to hedonic feeding through learning and context-driven mechanisms [85].

Specific projections from the IC to the ventral striatum mediate compulsive intake (that is, increase lever presses for food despite negative consequences) following intermittent access to palatable food, implicating this pathway in the transition from controlled to compulsive eating [86]. Using this model, it was also observed that heightened GLP-1- and PP (pancreatic peptide) with lower ghrelin characterized rats with the most compulsive-like eating. Additionally, the study also showed a correlative relationship between compulsive eating and both leptin and adiposity [86]. This suggests that insular activity may be sensitive to circulating metabolic hormones, which could contribute to binge eating behavior.

At the cellular level, animals prone to binge eating exhibit decreased excitability of leptin-sensitive pyramidal neurons in the anterior IC, suggesting that insular leptin signaling may normally suppress excessive intake and its impairment facilitates compulsive demand for food [87]. These findings suggest that leptin may modulate motivation or decision-making via a cortical circuit that is influenced by intermittent access to food. Importantly, recent work has demonstrated that direct interoceptive inputs from the brainstem to the IC shape learned feeding behavior by modulating the insula’s ability to associate internal state with external food cues via leptin receptor [88]. This interoceptive tuning of the IC may explain how bodily states like stress, satiety, or withdrawal influence learned food-seeking.

Additionally, endogenous opioids such as dynorphin act through an anterior IC to claustrum pathway to promote stress-induced binge eating, reinforcing the role of neuropeptide systems in modulating affective drives to eat [89]. In female rodents, a glutamatergic PVT to medial IC projection has been shown to specifically gate stress-induced binge eating, highlighting sex-specific neural mechanisms [90]. Overall, these data suggest that the insular cortex links internal physiological states and external food cues, integrating interoceptive, emotional, and cognitive signals to drive non-homeostatic feeding. Its involvement in context-driven cue reactivity, stress-induced binge episodes, and corticostriatal reward circuitry makes the IC a central node in the pathophysiology of binge eating.

#### Prefrontal Cortex

The prefrontal cortex (PFC) is a key cortical hub for executive control, decision-making, and reward valuation, and has emerged as a critical regulator of maladaptive feeding behaviors, including binge eating [91]. The PFC receives inputs from the thalamus, amygdala, hippocampus, and insular cortex, and sends projections to key subcortical structures involved in reward and motivation, including the NAc, lateral hypothalamus, lateral septum and periaqueductal gray [92, 93]. This extensive connectivity positions the PFC as a top-down regulator of both homeostatic and non-homeostatic feeding.

In binge eating models, dysregulated activity in the medial prefrontal cortex (mPFC) is commonly observed. Decreased mPFC activation correlates with loss of cognitive control and impulsivity in palatable food intake [91, 94]. In Anastasio et al., 2019 [94], motor impulsivity predicts vulnerability to binge-like eating in rats, and chemogenetic activation of ventral mPFC projections to the nucleus accumbens shell suppresses both impulsivity and high-fat food overconsumption, indicating this pathway functions as a neural “brake” on these behaviors. Supporting the idea of the prefrontal cortex as a region important for inhibitory control of feeding behaviors, optogenetic stimulation of the mPFC has been shown to suppress binge-like eating [95]. In this work, a subset of mPFC neurons that express vasoactive intestinal peptide (VIP) were identified and optogenetic activation of VIP + interneurons in the medial prefrontal cortex selectively suppressed binge-like intake of high-calorie palatable food without affecting chow consumption, with infralimbic VIP neurons reducing intake in a novelty- and value-dependent manner and prelimbic VIP neurons producing novelty-independent suppression, revealing that distinct VIPergic subpopulations modulate specific aspects of food reward-driven behavior. This functional specialization within PFC microcircuits suggests that discrete inhibitory networks contribute differently to binge eating, depending on whether the behavior is externally triggered or internally motivated.

The inhibitory influence of the PFC on feeding is mediated by multiple cell types and projection pathways. Activation of both inhibitory and glutamatergic PFC projections to the nucleus accumbens and lateral hypothalamus, respectively, can suppress binge eating and fat consumption [77, 91, 95, 96]. Some of these circuits have been shown to be sensitive to stressful stimuli and have an inhibitory effect on food intake and motivation to consume sucrose pellets [96]. This is particularly interesting as binge-eating behavior is mostly associated with episodes of stress [97, 98]. However, the PFC does not function solely as a “brake” on feeding as activation of PFC projections to the medial basolateral amygdala (mBLA), specifically via D1-expressing PFC neurons, can promote food intake [99]. These findings highlight the molecular and connectional diversity of the PFC and support the view that PFC neurons modulate behavior bidirectionally, either enhancing or diminishing cognitive control over emotionally and motor-related brain regions.

#### Translational Potential of Preclinical Animal Model Studies

Rodent binge‑eating models offer valuable insights into the neural mechanisms underlying compulsive intake by enabling controlled manipulation of feeding behavior in the absence of human-specific factors such as self-awareness or emotional distress. Many paradigms replicate key features of human binge eating, including rapid consumption of palatable food, self-control and impulsivity and increased motivation under intermittent access, which correspond to core aspects of binge‑eating disorder [21, 100]. These models have demonstrated that systemic or localized antagonism of opioid signaling, especially in the NAc, reduces binge‑like consumption, for example, µ‑opioid receptor antagonists diminished food-seeking and binge eating in rodents [21, 101–104]. Similarly, intermittent access to palatable food induces sensitization of dopaminergic systems, reinforcing compulsive intake patterns in a manner reminiscent of drug addiction [105, 106]. The translational value is further supported by parallel findings in humans. Neuroimaging studies show that individuals with BED exhibit reduced µ‑opioid receptor availability and dopamine synthesis capacity in the nucleus accumbens, mirroring rodent neuroadaptations [107]. Moreover, rodent studies reveal a shift from goal-directed to habit-like control over feeding, marked by increased dorsal striatal involvement, an analogous process observed in addictive behaviors [105]. Through these shared circuit-level adaptations, enhanced mesolimbic dopamine, opioid-mediated hedonic drive, and habit formation, rodent models of binge eating recapitulate fundamental neurobiological themes of addiction and thus offer robust translational relevance. Rodent models of binge eating also allow for precise dissection of genetic and pharmacological mechanisms that cannot be directly studied in humans. Single-gene manipulations in mice and rats have clarified how dopamine, opioid, and neuropeptidergic systems regulate compulsive food intake, providing causal links between molecular pathways and binge-like behavior. These models have also been essential for evaluating potential pharmacotherapies [107]. For example, lisdexamfetamine (LDX), now FDA-approved for BED, was shown in female rat models to selectively reduce chocolate bingeing without altering chow intake, an effect mediated through α1-adrenergic and D1 receptor activity [108, 109]. Other compounds, including methylphenidate, selective serotonin reuptake inhibitors (SSRIs), 5-HT2C receptor agonists, and monoamine stabilizers, have all reduced binge-like episodes in rodents, highlighting dopaminergic and serotonergic pathways as therapeutic targets [25, 107, 109–113]. Neuropeptidergic systems such as the opioid, orexin, GABAergic, and nociceptin/orphanin FQ pathways have also been implicated through antagonist studies, while novel targets such as TAAR1 and sigma1 receptors have emerged from preclinical testing [114, 115].

The recent success of glucagon-like pepetide-1 receptor (GLP1R) agonists as therapeutic agents underscores the important role of preclinical research in this area. Some of the first rodent studies on GLP1R described strong effects on reducing food intake [116, 117]. Rodent models have demonstrated a key role for the GLP-1 system in mediating and controlling binge-like behavior, including decreased GLP-1 following binge-like behavior, as well as specific decreases of palatable food following GLP-1 administration [78, 86, 118–121]. These findings have now been effectively translated to clinical populations [122, 123], although larger placebo-controlled studies are needed to fully establish the effectiveness compared to obesity. These new developments underscore the importance of animal model work in identifying and characterizing novel therapeutic targets.

Together, these findings underscore how rodent models bridge mechanistic insights with therapeutic discovery, offering a platform to screen drugs, clarify receptor-level actions, and identify neurobiological substrates that can be targeted in patients with BED.

## Conclusion

Rodent binge eating models have revealed key neural and molecular mechanisms of compulsive intake, showing parallels with addiction. Paradigms using limited access, stress, or cues implicate the nucleus accumbens, prefrontal cortex, insula, and hypothalamus in binge behavior, with dopamine, opioid, orexin, and other several neuropeptide systems shaping reward and control. These models also mirror human findings, enabling precise genetic and pharmacological testing that would not be possible in human BED patients. This translational bridge has already guided therapy, exemplified by lisdexamfetamine, and continues to inform novel strategies. Together, rodent models remain powerful tools for uncovering mechanisms and advancing treatments for BED.

## Data Availability

No datasets were generated or analysed during the current study.
